# Paired Associative Electroacupuncture and Transcranial Magnetic Stimulation in Humans

**DOI:** 10.3389/fnhum.2019.00049

**Published:** 2019-02-12

**Authors:** Yi Huang, Jui-Cheng Chen, Chun-Ming Chen, Chon-Haw Tsai, Ming-Kuei Lu

**Affiliations:** ^1^Graduate Institute of Biomedical Sciences, Medical College, China Medical University, Taichung, Taiwan; ^2^Neuroscience Laboratory, Department of Neurology, China Medical University Hospital, Taichung, Taiwan; ^3^School of Medicine, Medical College, China Medical University, Taichung, Taiwan; ^4^Department of Radiology, China Medical University Hospital, Taichung, Taiwan

**Keywords:** electroacupuncture, motor cortex, motor evoked potential, paired associative stimulation, transcranial magnetic stimulation

## Abstract

Pairing transcutaneous electric nerve stimulation (TENS) and transcranial magnetic stimulation (TMS) with specific stimulus-intervals induces associative motor plasticity at the primary motor cortex (M1). Electroacupuncture (EA) is an established medical technique in the eastern countries. This study investigates whether EA paired with TMS induces distinct M1 motor plasticity. Fifteen healthy, right-handed subjects (aged 23.6 ± 2.0 years, eight women) were studied. Two-hundred and twenty-five pairs of TMS of the left M1 preceded by right EA at acupoint “Neiguan” [Pericardium 6 (PC6), located 2 decimeters proximal from the wrist wrinkle] were respectively applied with the interstimulus interval (ISI) of individual somatosensory evoked potential (SSEP) N20 latency plus 2 ms (N20+2) and minus 5 ms (N20-5) with at least 1-week interval. The paired stimulation was delivered at a rate of 0.25 Hz. Sham TMS with a sham coil was adopted to examine the low-frequency EA influence on M1 in eleven subjects. M1 excitability was assessed by motor-evoked potential (MEP) recruitment curve with five TMS intensity levels, short-interval intracortical inhibition (SICI), intracortical facilitation (ICF) and cerebellar inhibition (CBI) at the abductor pollicis brevis (APB) muscle of the right hand before and after the EA-M1 paired associative stimulation (PAS). In addition, median nerve SSEPs and H-reflex were respectively measured to monitor somatosensory and spinal excitability. The MEP showed significantly facilitated after the sham EA-M1 PAS while tested with 80% of the TMS intensity producing on average 1 mV amplitude (i.e., MEP_1 mV_) in the resting APB muscle. It was also facilitated while tested with 90% MEP_1 mV_ irrespective of the stimulation conditions. The SSEP showed a higher amplitude from the real EA-M1 PAS compared to that from the sham EA-M1 PAS. No significant change was found on SICI, ICF, CBI and H-reflex. Findings suggest that repetitive low frequency EA paired with real TMS did not induce spike-timing dependent motor plasticity but EA paired with sham TMS induced specific M1 excitability change. Complex sensory afferents with dispersed time locked to the sensorimotor cortical area could hamper instead of enhancing the induction of the spike-timing dependent plasticity (STDP) in M1.

## Introduction

Primary motor cortex (M1) receives multi-direction gated sensory information and executes the final motor command in humans (Cheng et al., 2017; Lei et al., 2018). It plays a key role in motor learning and motor performance. Nowadays there are several non-invasive techniques capable of inducing M1 excitability change, such as fixed-frequency repetitive transcranial magnetic stimulation (rTMS), paired associative stimulation (PAS; Stefan et al., 2000), theta burst stimulation (Huang et al., 2005) and so on (for a review see Quartarone et al., 2006; Huang et al., 2017). The modification of abnormal M1 excitability by these non-invasive brain stimulation techniques has shown possible clinical benefits on neuropathic pain and Parkinson’s disease (Lefaucheur et al., 2012; Brys et al., 2016; Goodwill et al., 2017).

PAS is one of the well-known non-invasive brain stimulation techniques with which to investigate Hebbian principles of neural plasticity in humans (Stefan et al., 2000). The most traditional form of PAS consists of repeated pairing of a single electric stimulus at the peripheral median nerve and a TMS pulse on the contralateral M1 with a specific interstimulus interval (ISI) between these two stimuli. It induces aftereffects representing the associative long-term potentiation (LTP)- and depression (LTD)-like phenomenon that bears resemblance to spike-timing dependent plasticity (STDP) as it has been elaborated in animal models (Carson and Kennedy, 2013). An ISI of 25 ms or individual N20 latency plus 2 ms (N20+2) results in arrival of the afferent sensory signal elicited by the peripheral median nerve stimulation before or almost at the same time in M1 when the TMS of the M1 generates actions potentials in excitatory interneurons and corticospinal neurons. The order of the events in M1 is reversed if an ISI of 10 ms or individual N20 latency minus 5 ms (N20-5) is applied (Müller-Dahlhaus et al., 2010). Nevertheless, the PAS effect can be affected by many factors and identifying these factors is a challenge in the current clinical practice (Murase et al., 2015; Huang et al., 2017). Among the factors relevant to the PAS effects, attention and the afferent somatosensory stimulation play an instrumental role in determining the magnitude of the PAS effects (Carson and Kennedy, 2013). In the original PAS protocol, the afferent somatosensory input is mediated through transcutaneous electric nerve stimulation (TENS) at wrist median nerve. The electric stimulation is prone to be adapted for most of the subjects so an additional device for maintaining the subject’s attention during the experiment is usually required (Stefan et al., 2004; Lu et al., 2009).

Acupuncture is an ancient medical technique frequently applied for pain control in the eastern countries. Evidences from functional brain imaging have suggested that acupuncture needle stimulation actually modulates specific neural networks in the brain (Hui et al., 2000; Fang et al., 2009), and different acupuncture modalities recruit different brain networks (Jiang et al., 2013). The invention of electric power allows acupuncture delivering a repetitive and constant stimulation at the specific stimulation site which is called “acupoint.” Stimulation at different acupoints may have distinct associations with neurocircuits. For example, acupuncture at acupoint “Shenmen; HT7,” but not “Neiguan; PC6,” improves the ventral tegmental area (VTA)-nucleus accumbens dopaminergic function *via* inhibition of brain-derived neurotrophic factor (BDNF) expression in the VTA (Zhao et al., 2015). Intriguingly, the acupoint “Neiguan; PC6” is approximately located in the same site where median nerve TENS is adopted for PAS. PC6 has been documented with a capacity to change brain activation patterns relevant to attention and improve cognition for stroke patients (Chou et al., 2009; Jung et al., 2015). A study compared the median nerve somatosensory evoked potentials (SSEPs) between TENS, electroacupuncture (EA) and sham stimulation of the specific acupoints (i.e., ST36 and ST37) in the leg (Kang et al., 2015). The results showed that EA, but not TENS nor sham stimulation, alters the mean amplitude of N20 and N30 during and post the stimulation periods (Kang et al., 2015). The findings also suggest a possibility that EA may have different influences on the somatosensory cortex from TENS stimulation. In a recent study, recruitment of additional corticospinal pathway has been achieved by the state-dependent PAS protocol in which sensorimotor event-related desynchronization (ERD) of the β-band was used to trigger peripheral stimulation (Kraus et al., 2018). Gathering evidences raise an issue: whether the peripheral EA stimulation carries a distinct impact on the PAS effect? If the answer is yes, in which level the influence may occur? Since the central mechanism of EA remains not clear, we wondered that EA paired with TMS has a consistent STDP-like effect similar to the traditional PAS. Complex sensory afferents might disrupt instead of enhancing STDP in M1. This study aims to clarify this issue.

## Materials and Methods

### Subjects

In total 15 right-handed (Oldfield, 1971) healthy subjects (23.6 ± 2.0 years, eight women) were recruited in this study. They all received both real and sham stimulation conditions including two different stimulation protocols in each [see “Paired Associative Electroacupuncture and TMS (PAET)” section]. This study was carried out in accordance with the recommendations of the local ethics committee of the China Medical University Hospital with written informed consent from all subjects. All subjects gave written informed consent in accordance with the Declaration of Helsinki. The protocol was approved by the local ethics committee of the China Medical University Hospital (CMUH104-REC2-164). They all received a brain MRI examination to exclude structure lesion.

### Procedures

#### Measurement of Motor Cortical Excitability

TMS was delivered through a focal figure-of-eight stimulating coil (inner diameter of each wing, 70 mm) connected *via* a BiStim module to two Magstim 200 magnetic stimulators (Magstim Co., Carmarthenshire, Wales, UK). The optimal coil position (“hot spot”; M1_HAND_) is determined as the site where TMS at a slightly supra-threshold intensity produced consistently the largest motor-evoked potentials (MEPs) in the right abductor pollicis brevis (APB). The intensity of TMS adjusted to produce MEPs of on average 1 mV in peak-to-peak amplitude in the resting APB is defined as the MEP_1 mV_. The individual resting motor threshold (RMT) and active motor threshold (AMT) was determined over the left M1_HAND_. AMT was additionally determined over the inion (inion AMT) prior to the baseline recording. The detailed procedure for determining RMT and AMT has been described elsewhere (Lu et al., 2012).

MEP IO curve is a quantitative measurement for corticospinal excitability. It was measured by stimulation at five intensity levels ranging from two levels below and two levels above MEP_1 mv_ with a step of 20% MEP_1 mv_ (i.e., five levels of stimulus intensity). Due to the constraint of the whole experiment time, eight stimuli were recorded at each intensity level. The ISI was determined as 7 s with a 25% variance to limit the anticipation effect.

#### Short-Interval Intracortical Inhibition (SICI)/Intracortical Facilitation (ICF)

Short-interval intracortical inhibition (SICI) and intracortical facilitation (ICF) were studied using an established paired-pulse TMS protocol (Kujirai et al., 1993; Ziemann et al., 1996). In brief, the two magnetic stimuli were given through the same figure-of-eight stimulating coil over the left M1_HAND_ and the effect of the sub-threshold conditioning stimulus on the test MEP elicited by the subsequent supra-threshold test stimulus (TS) was investigated. SICI was assessed at an ISI of 2.0 ms because at this interval SICI is not contaminated by SICF (Peurala et al., 2008). At the baseline recording, the condition stimulation (CS) intensity was adjusted to produce approximately 50% inhibition in order to provide highest sensitivity for detection of changes in SICI after PAS. The CS intensities usually ranged from 70 to 90% AMT in different individuals (Lu et al., 2012). This CS intensity was kept constant throughout the experiment. ICF was assessed at an ISI of 10 ms (Ziemann et al., 1996). The CS intensities usually ranged from 75 to 95% AMT in different individuals to produce consistent test MEP facilitation (Ziemann et al., 1996; Lu et al., 2012).

#### Cerebello-Motor Cortical Inhibition (CBI)

Cerebellar inhibition (CBI) was measured with a double-cone coil positioned at the midpoint of the inion and the right incisura mastoidea for CS (Ugawa et al., 1995). 95% AMT_inion_ was adopted for the CS intensity to avoid any corticospinal excitability (Ugawa et al., 1995; Lu et al., 2012). The TS was delivered at left M1 and kept an intensity to elicit an average MEP of ~0.7 mV while delivered alone. The ISI between CS and TS was 6 ms.

#### Median Nerve Somatosensory-Evoked Potentials (SSEPs)

The early component of median nerve SSEP (N20-P25) is an index of the somatosensory cortex (S1) excitability. It was recorded while the subjects voluntarily relaxed with eyes closed (Krivanekova et al., 2011). The active electroencephalography electrode was placed at C3’, 2 cm posterior to C3 according to the International 10–20 system, corresponding to the putative site of the left S1. The reference electrode was placed on the frontal midline (Fz). The right median nerve was stimulated through a bipolar electrode (cathode proximal) with a constant current square pulse of 0.2 ms duration at rate of 3 Hz (Digitimer DS7A, Digitimer Ltd., UK). Stimulus intensity is adjusted to 110% of twitching threshold in the right thenar muscle. Six-hundred trials were recorded and averaged for offline analysis.

#### H-Reflex

The H-reflex is an electrical analog of the spinal stretch reflex and an index of spinal excitability. Subjects were positioned in sitting with the elbow at 45° flexion. A stimulus location in the medial bicipital groove was determined where a maximal H-reflex without an M-wave was evoked on the flexor carpi radialis muscle. Stimulation intensity was sequentially increased with a step of 1 mA from a sub-H-reflex threshold intensity to the level when H-reflex amplitude began to decline. The intensity which provokes a maximal H-wave peak-to-peak amplitude (H_Max_) was determined and used for the following measurement.

#### Acupuncture

**Figure 1:**
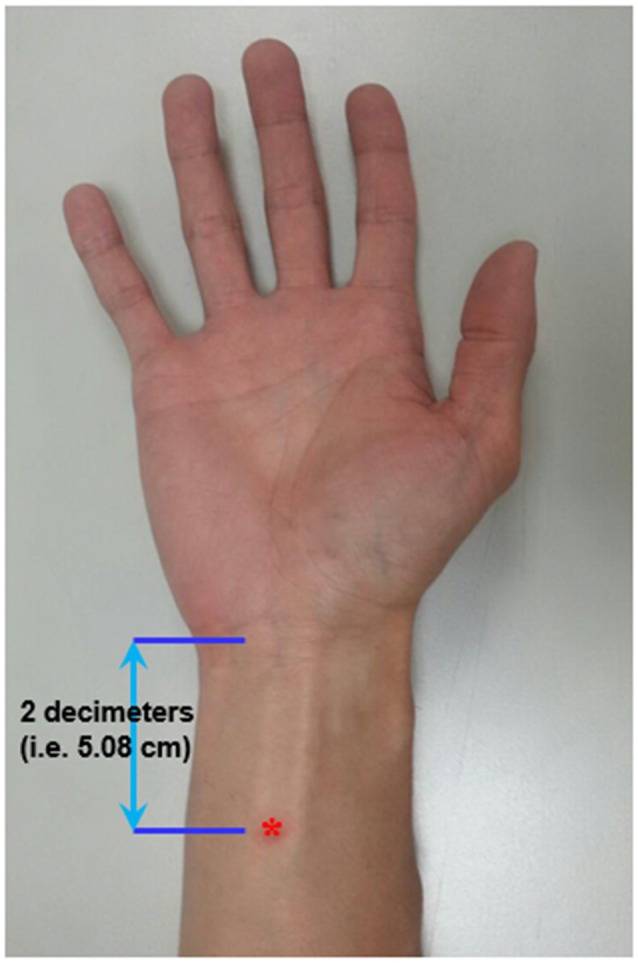
Localization of the acupoint “Neiguan” (Pericardium 6, PC6; *).

Acupoint “Neiguan” (Pericardium 6; PC6) is located 2 decimeters (i.e., 5.08 cm) proximal from the wrist wrinkle (Chou et al., 2009; Figure 1). This location is approximately the same site where median nerve SSEP is stimulated. Prior to the EA, an acupuncture needle (sterile stainless needle, 0.3 mm diameter, 50 mm length, Yuguang Corporation, Taiwan) was inserted into the right PC6 with a depth of 10 mm. The *de-chi* sensation of the acupuncture is somewhat subjective and difficult to be quantitatively defined. Therefore, it was not essential for subjects to report *de-chi* sensation in this study.

#### Paired Associative Electroacupuncture and TMS (PAET)

**Figure 2:**
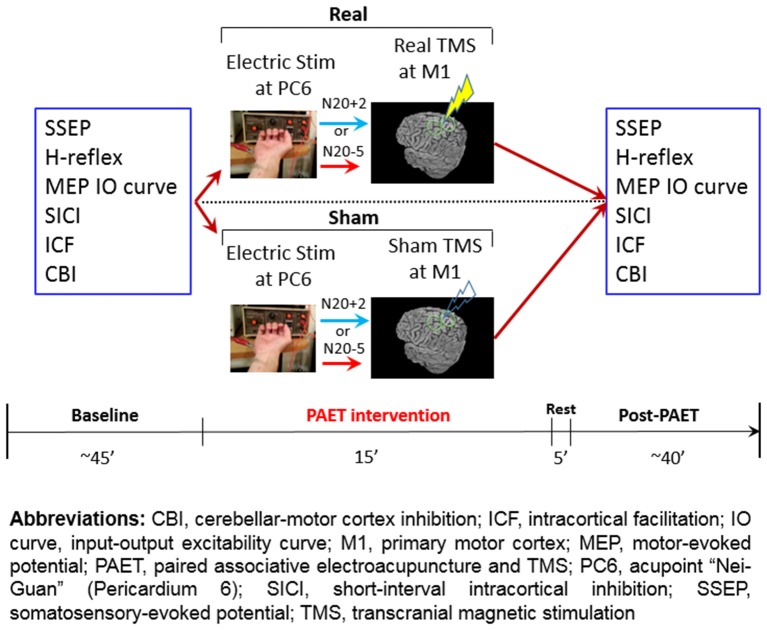
Experimental design and the time line of the paired associative electroacupuncture and transcranial magnetic stimulation (TMS; PAET) study.

The conventional PAS protocol consists of 225 pairs of a cutaneous electric stimulation of the median nerve at the wrist followed by a single TMS at the contralateral M1_HAND_ (Stefan et al., 2000). The cutaneous nerve stimulation was replaced by the EA at PC6 in the current PAET paradigm. The needle for EA was connected to the stimulator (Digitimer DS7A, Digitimer Ltd., UK) which was triggered by a computer-based interface (Spike2 for Windows, Version 3.05, CED, UK). The stimulus intensity was adjusted to evoke a same level of the right-hand muscle twitching observed during the SSEP recording. There were two intervals between the EA and the TMS, individual N20 latency plus 2 ms (N_20+2_) and minus 5 ms (N_20–5_). Based on the STDP principle and the previous evidences, the N_20+2_ interval produces LTP-like and the N_20–5_ interval produces LTD-like plasticity (Ziemann et al., 2004; Müller-Dahlhaus et al., 2010; Lu et al., 2016). The intensity of TMS was adjusted to produce MEPs of on average 1 mV in peak-to-peak amplitude in the resting APB. To carefully examine the effect of the repetitive low-frequency EA stimulation, we designed sham TMS by using a sham TMS coil (Magstim Co., Carmarthenshire, Wales, UK). In total 225 pairs were delivered at a rate of 0.25 Hz (i.e., duration of PAET, 15 min; Figure 2).

### Statistical Analysis

Repeated measures analyses of variance (rmANOVA) was applied to test the effects of PAET on the five intensity steps of the MEP, SICI, ICF, CBI, SSEP and H-reflex. The within-subject effects were TIME (Pre-PAET vs. Post-PAET), PROTOCOL (PAET_N20+2_ vs. PAET_N20-5_) and CONDITION (Real vs. Sham). Conditional on a significant *F* value, *post hoc* comparisons were performed using paired-sample *t*-tests with Bonferroni’s correction. Violation of sphericity was checked with Mauchly’s test and degrees of freedom were adjusted whenever Mauchly’s *W* < 0.05 using the Greenhouse-Geisser correction (SPSS 22.0). Data are reported as means ± SD if not stated otherwise.

## Results

All of the subjects were cooperative throughout the experimental procedures. None of them reported any noticeable adverse effects during or after the study.

The mean RMT, AMT and inion AMT of the 15 participants were 54 ± 7.9%, 45 ± 7.0% and 39 ± 4.8%, respectively. The mean MEP_1 mv_ was 65 ± 12.0%. The intensities applied for measuring SICI, ICF and CBI were listed in Table 1. We analyzed the data of the 15 subjects for three main effects (i.e., CONDITION, PROTOCOL and TIME). RmANOVA of the MEP amplitude revealed a significant effect of TIME (*F*_(1,14)_ = 8.8, *P* = 0.01) and a significant CONDITION × TIME interaction at TMS intensity of 80% MEP_1 mV_ (*F*_(1,14)_ = 5.63, *P* = 0.03), and a significant effect of TIME at TMS intensity of 90% MEP_1 mV_ (*F*_(1,14)_ = 12.73, *P* = 0.003; Table 2). RmANOVA of the SICI did not show any significant effect (all *P* > 0.08). A significant PROTOCOL × TIME interaction was found for the analysis of ICF (*F*_(1,14)_ = 7.46, *P* = 0.02). A significant main effect of CONDITION was found on SSEP (*F*_(1,14)_ = 8.97, *P* = 0.01). RmANOVA of the H_Max_ did not show any significant effect (all *P* > 0.3). The statistical power reaches 0.93 with the effect size of 0.4 for the three-way rmANOVA.

**Table 1:** The transcranial magnetic stimulation (TMS) intensity* adopted for measuring short-interval intracortical inhibition (SICI), intracortical facilitation (ICF) and cerebello-motor cortical inhibition (CBI).

	SICI	ICF	CBI
Conditioning	43 ± 7	43 ± 7	37 ± 5
Test	68 ± 12	69 ± 12	63 ± 12

*Presented as percentage of maximal stimulator output.

**Table 2:** Repeated measures analysis of variance (rmANOVA) of the paired associative electroacupuncture (EA) and TMS (PAET) effect.

		MEP										SICI		ICF		CBI		SSEP		*H*_Max_	
		80%		90%		100%		110%		120%											
	*d.f*.	*F*	*P*	*F*	*P*	*F*	*P*	*F*	*P*	*F*	*P*	*F*	*P*	*F*	*P*	*F*	*P*	*F*	*P*	*F*	*P*
Condition^a^	1, 14	1.96	0.18	1.93	0.19	4.38	0.06	2.15	0.17	1.40	0.26	0.94	0.35	0.04	0.85	2.09	0.17	**8.97**	0.01*	0.66	0.44
Protocol^b^	1, 14	0.31	0.58	0.82	0.38	0.06	0.81	0.002	0.97	0.99	0.34	0.09	0.77	3.62	0.08	0.96	0.35	0.02	0.88	0.07	0.79
Time^c^	1, 14	**8.80**	0.01*	**12.73**	0.003**	0.65	0.44	3.69	0.08	0.59	0.46	3.45	0.09	1.85	0.20	2.61	0.13	2.75	0.12	0.95	0.35
Condition × Protocol	1, 14	0.60	0.45	0.37	0.55	0.14	0.72	0.96	0.34	2.08	0.17	1.33	0.27	0.83	0.38	2.35	0.15	1.92	0.19	0.17	0.69
Condition × Time	1, 14	**5.63**	0.03*	4.04	0.06	4.09	0.06	2.26	0.16	0.04	0.85	0.007	0.94	0.14	0.72	1.36	0.26	0.01	0.92	0.06	0.81
Protocol × Time	1, 14	0.28	0.61	0.88	0.36	0.46	0.51	0.02	0.90	1.55	0.23	0.29	0.60	**7.46**	0.02*	0.37	0.55	1.87	0.19	0.30	0.60
Condition × Protocol × Time	1, 14	0.48	0.50	1.47	0.25	0.37	0.55	0.33	0.57	0.25	0.63	1.26	0.28	0.05	0.83	2.43	0.14	0.47	0.51	0.86	0.38

*P < 0.05, **P < 0.01. ^a^2 levels (Real and Sham). ^b^2 levels (N20+2 and N20-5). ^c^2 levels (pre-PAET and post-PAET). Abbreviations: CBI, cerebellar inhibition; d.f., degrees of freedom; H_Max_, maximal H-wave peak-to-peak amplitude; ICF, intracortical facilitation; MEP, motor evoked potential; SICI, short-interval intracortical inhibition; SSEP, somatosensory evoked potential.

**Figure 3:**
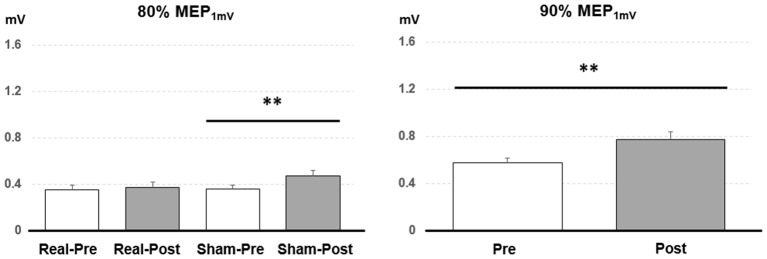
*Post hoc* comparisons of the motor evoked potential (MEP) evoked with different TMS intensities for the 15 subjects. MEP_1 mV_ means the TMS intensity inducing on average 1 mV MEP amplitude in the resting abductor pollicis brevis (APB) muscle. The MEP amplitude showed a significant facilitation after the sham PAET intervention with 80% MEP_1 mV_ and the PAET intervention with 90% MEP_1 mV_. Data are shown by means ± standard errors. **P < 0.01 by paired *t*-test with Bonferroni’s correction.

**Figure 4:**
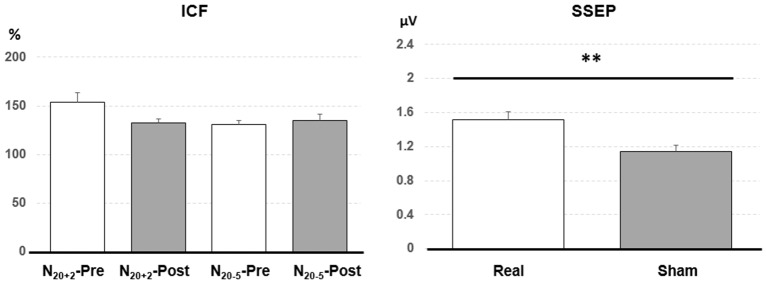
*Post hoc* comparisons of the intracortical facilitation (ICF) and somatosensory evoked potential (SSEP). The ICF comparisons did not show any significant difference. The SSEP amplitude of the real condition was significantly higher than that of the sham condition. Data are shown by means ± standard errors. **P < 0.01 by paired *t*-test with Bonferroni’s correction.

*Post hoc* comparisons showed a significant MEP amplitude increase after Sham condition at 80% MEP_1 mV_ (Pre/Post: 0.36 ± 0.16/0.47 ± 0.25 mV, *P* < 0.01 by paired *t*-test; Figure 3). At 90% MEP_1 mV_, the MEP amplitude showed a significant increase after the PAET intervention irrespective of CONDITION and PROTOCOL (Pre/Post: 0.58 ± 0.26/0.77 ± 0.49 mV, *P* < 0.01 by paired *t*-test; Figure 3). There was no significant difference from the *post hoc* comparisons on ICF (all *P* > 0.05 by paired *t*-test; Figure 4). The *post hoc* comparison of SSEP revealed a significant difference between the real condition and the sham condition (Real/Sham: 1.5 ± 0.8/1.1 ± 0.6 μV, *P* < 0.01 by paired *t*-test; Figure 4).

## Discussion

### PAET Effect on M1 Excitability

The current data support the notion that the PAET intervention may have complex influences on the M1 excitability. While M1 excitability was investigated with a relatively low TMS intensity such as 80% or 90% MEP_1 mV_, the PAET, particularly the sham PAET, significantly facilitated the MEP amplitude. Following the increase of the TMS test intensity, the aftereffect of PAET was not detectable with the MEP recording.

It has been known that acupuncture has clinical effects on analgesia (Xiang et al., 2017; Pang et al., 2018). The rTMS protocols which facilitate M1 excitability also revealed a benefit response on neuropathic pain (Hosomi et al., 2013; Boyer et al., 2014). Whether the analgesic effect of acupuncture is mediated through a similar mechanism with rTMS remains not clear and needs further investigation. Our findings on the sham PAET provided evidence showing that EA may facilitate M1 excitability and the change of the M1 excitability is only detectable with a low to moderate TMS intensity. In addition to EA, the trains of 1 Hz electrical stimulation and 25 Hz repetitive magnetic stimulation on the peripheral nerve also have shown ability to modulate sensorimotor excitability (Kaelin-Lang et al., 2002; Gallasch et al., 2015). Since the low intensity TMS activates interneurons instead of corticospinal neurons (Di Lazzaro and Rothwell, 2014), it is likely that the PAET-induced M1 plasticity is mediated through the interneurons. SICI and ICF are two common parameters representing the function of the interneurons (Chen et al., 1998).

The reason why the sham PAET induced a higher MEP amplitude compared to the real PAET can be explained by the frequency of the repetitive TMS. In this study the real PAET consists of 225 TMS pulses with 0.25 Hz. It has been known that the supra-threshold rTMS of low frequency (i.e., 1 Hz or less) at M1 suppresses corticospinal excitability and reduced MEPs (Chen et al., 1997). It might be achieved by suppressing the amplitude of later I-waves, which have been proposed based on the epidural observation (Di Lazzaro et al., 2008). The reduction of the corticospinal neuronal excitability was supposed to be robust enough to block or erase the EA-induced interneuron plasticity mentioned above.

The current findings suggest that there is no significant role on the protocol difference. That is, we did not find any M1 excitability change following the spike-timing dependent pattern. One of the possible explanations is that EA carries complex sensory modalities including somatosensory, pain and variable attention. These different sensory afferents are actually not homogeneously time-locked to the cortical sensorimotor area. Despite the EA location (i.e., PC6) is very close to that adopted for the traditional PAS, the subtle difference of the location between these two methods could result in different findings. Therefore, it would be difficult to detect typical M1 motor plasticity similar to that induced by the traditional PAS protocols. Another possibility for the absence of a significant spike-timing dependent pattern is probably due to the inter-subjects variability, which has been recognized as a notable issue for the non-invasive brain stimulation protocols including PAS (López-Alonso et al., 2014; Guerra et al., 2017).

### SICI/ICF

The current findings on SICI are agree with the previous report which revealed SICI was not consistently affected by the traditional PAS intervention (Russmann et al., 2009). SICI has been found mediated by the GABA_A_ receptors (Di Lazzaro et al., 2006; Florian et al., 2008). In a recent animal study, EA may increase GABA_A_ receptors but the influence is only restricted in the spinal cord (Jiang et al., 2018).

The PAS protocol facilitating M1 excitability increases ICF, which suggests that the modification of M1 excitability is contributed from the intracortical excitatory circuits (Pyndt and Ridding, 2004). In the *post hoc* analysis we could not find any significant change following the PAET (Figure 4). A further study with more conditioning and test TMS intensities would be helpful to clarify the role of ICF in the M1 excitability change induced by the PAET.

### SSEP/H-Reflex

Low frequency (0.05 Hz) EA intervention for 4 months can induce fluctuating cortical plasticity in the somatosensory area, pain-related areas and limbic/paralimbic areas in rats (Wu et al., 2018). In the previous report the amplitude of the median SSEP was found significantly increased by 2 Hz EA at the leg acupoint for 15 min (Kang et al., 2015). However, the SSEP finding in this study did not support the notion that the EA *per se* induce a significant plasticity in the somatosensory area. The main effect of CONDITION instead of TIME from the current rmANOVA analysis suggests that EA paired with real or sham M1 TMS may induce different excitability in the somatosensory area (Table 2, Figure 4). The finding is consistent with the previous report showing that the traditional PAS consisting of paired median nerve stimulation and TMS at M1 changes SSEP (Murakami et al., 2008). Since the SSEP amplitude may depend on the adopted method (Macerollo et al., 2018), a more sensitive method such as using a non-cephalic reference would be better to measure the excitability change of the somatosensory area in this regard.

H-reflex represents the spinal excitability. Fifty hertz EA can modulate H-reflex response in the experimental rat (Escobar-Corona et al., 2017). The current low frequency (i.e., 0.25 Hz) EA did not significantly alter H-reflex, suggesting that the observed MEP changes are induced in the cortical level, probably the M1 instead of the spinal cord.

There are limitations in this study. As a proof-of-concept study, we merely recruited a limited number of subjects. This inevitably constrained our findings and interpretations. In addition, there was no control condition (e.g., sham or TENS) for the EA stimulation. This also diminishes the significance of the current findings. Nevertheless, the method adopted in this study could shed light on how to combine EA and TMS approaches in the future.

## Conclusion

Repetitive EA paired with sham TMS at M1 induces specific motor cortical plasticity which could be only detectable with moderate TMS intensities. EA paired with real low-frequency (i.e., 0.25 Hz) rTMS may interrupt instead of enhancing this kind of plasticity. That is, there is no spike-timing dependent character for this plasticity. Complex sensory afferents with dispersed time locked to the sensorimotor cortical area may hamper the induction of the STDP-like plasticity in M1. GABAergic, glutamatergic, cerebellar afferent and spinal excitability respectively examined by SICI, ICF, CBI and H-reflex were not significantly affected.

## Data Availability

All datasets generated for this study are included in the manuscript.

## Author Contributions

YH: subject recruitment, acquisition of data, writing first draft of the manuscript. J-CC: experimental design, critical review of the manuscript, revision of the first draft. C-MC: experimental execution, acquisition of data, review of the manuscript. C-HT: experimental design, critical review of the manuscript, comments on the manuscript. M-KL: study concept and experimental design, data analysis and interpretation, critical revision of the manuscript.

## Conflict of Interest Statement

The authors declare that the research was conducted in the absence of any commercial or financial relationships that could be construed as a potential conflict of interest.
